# Determinants of peri-operative blood transfusion in a contemporary series of open prostatectomy for benign prostate hyperplasia

**DOI:** 10.1186/s12894-016-0134-x

**Published:** 2016-03-28

**Authors:** Mathew Y. Kyei, George O. Klufio, James E. Mensah, Samuel Gepi-Attee, Kwabena Ampadu, Bernard Toboh, Edward D. Yeboah

**Affiliations:** Department of Surgery and Urology, School of Medicine and Dentistry, College of Health Sciences, University of Ghana, P. O. Box 4236, Accra, Ghana

## Abstract

**Background:**

The objective of this study was to determine the factors responsible for peri-operative blood transfusion in a contemporary series of open prostatectomy for benign prostate hyperplasia and thus offer a guide for blood product management for the procedure.

**Methods:**

This was a prospective study of 200 consecutive patients who underwent open prostatectomy for BPH from January 2010 to September 2013 at the Korle Bu Teaching Hospital, Accra. The data analyzed included the pre-operative blood haemoglobin level (Hb), presence of co-morbidities, the case type, indication for the surgery, ASA score, anaesthetic method used, systolic blood pressure, status of the operating surgeon, duration of surgery and the operative prostate weight. The transfusion of blood peri-operatively was also documented.

**Results:**

The mean age of the patients was 69.1 years. Elective cases formed 83.5 % with refractory retention of urine being the commonest indication for surgery (68.0 %). The mean pre-operative Hb was 12.1 g/dl. Consultants performed 56.0 % of the prostatectomies. Transvesical approach was used in 90.0 % of the cases. The mean operative time was 101.3mins (range 35.0–240.0) with a mean operative prostate weight of 110.8 g (range 15–550 g). Most of the patients (82.0 %) had spinal anaesthesia. The blood transfusion rate was 23.5 %. The transfusion rate was significantly higher in patients with anaemia (*p* = .000), emergency cases (*p* = .000), the use of general anaesthesia (*p* = .002), a resident as the operating surgeons (*p* = .034), prostate weight >100 g (*p* = .000) and duration of surgery (*p* = .011). In a multivariable logistic regression analysis however only the pre-operative Hb (*p* = .000. OR 0.95, 95 % CI [0.035–0.257]) and the duration of surgery (*p* = .025, OR 1.021, 95 % CI [1.003–1.039]) could predict blood transfusion in open prostatectomy for BPH in this series.

**Conclusions:**

A ‘group and save’ policy should be the preferred blood ordering procedure for patients with Hb ≥ 13.0 g/dl scheduled for an elective open prostatectomy for BPH under spinal anaesthesia. A long operative time however may increase the need for blood transfusion.

## Background

Open prostatectomy for the surgical management of benign prostate hyperplasia (BPH) is now rarely used in most developed countries except for large prostates [1, 2]. This exclusive indication for the procedure in these countries is currently being shared with newer modalities of treatment like the holmium laser resection of the prostate [3]. However in resource poor countries, open prostatectomy remains the main mode of surgical management of benign prostate hyperplasia irrespective of the prostate size [4]. The surgical outcomes of open prostatectomy for BPH continue to be the basis for comparing and evaluating the effectiveness and safety of the newer methods of surgical management of large prostates [5]. This is because of the excellent clinical outcomes with improvement in lower urinary tract symptoms and an observed lower failure rate [5]. Thus a report on contemporary series of open prostatectomy in this era of newer treatment modalities may provide useful information in relation to blood transfusion requirements as we evaluate these newer techniques.

A frequently encountered challenge in open prostatectomy compared to the newer methods of surgical management of BPH is peri-operative bleeding requiring blood transfusion. The reported blood transfusion rates in open prostatectomy for BPH range from 3.3 % to 36.8 % [2, 5–8]. Blood transfusion carries risk of transfusion reactions and disease dissemination [9, 10] and there is substantial economic cost associated with allogeneic transfusions [11]. In developing countries where open prostatectomy is currently mostly practiced, assess to blood for transfusion is limited as there are only few voluntary blood donors. These surgeries are sometimes unduly delayed on account of lack of acceptable blood donors. An understanding of the factors that determine the transfusion rate in a contemporary series of open prostatectomy for BPH may form the basis for deciding pre-operatively, the likelihood of needing blood transfusion in a particular operation. This will enable a more rational ordering of blood products for the procedure as part of a blood management strategy. It will also bring to light modifiable factors that when addressed, could potentially make the transfusion rate in open prostatectomy comparable to the newer minimally invasive methods of prostatectomy for BPH.

## Methods

This was a prospective study on the management of 200 consecutive patients who had consented in writing to undergo open prostatectomy for benign prostate hyperplasia at the Urology Unit of the Korle Bu Teaching Hospital in Accra. The study period was from January 2010 to September 2013 and approved by the Ethical and Protocol committee of the Korle Bu Teaching Hospital under the Medical Directorate. The diagnosis of BPH was made on the basis of the patient’s clinical presentation and the finding of a prostate with benign features on digital rectal examination. Patients noted to have abnormal digital rectal examination or elevated total PSA had transrectal ultrasound guided prostate biopsy (12 cores) for histological confirmation of BPH.

Four consultant urologists and three senior urology residents carried out the operations in this study. The total number of open prostatectomy for BPH that had been performed by the consultants prior to the study ranged from 80–600 while that of the residents was 10–25 cases.

The surgical method used was either a transvesical open prostatectomy or Millins retropubic prostatectomy as described in Campbell Urology 7^th^ Edition (2007) [12].

For the technique of the transvesical prostatectomy, the patient is positioned in the supine position and the suprapubic area shaved. The lower abdomen and external genital area are prepped and draped. A 22 or 24-Fr Foley urethra catheter is inserted into the bladder and the bladder filled with 250 mL of saline and the catheter removed. A lower midline incision is made and deepened through the subcutaneous tissue. The linea alba is incised, and the rectus abdominis muscles separated in the midline. The transversalis fascia is incised to expose the space of Retzius. The peritoneum is swept cephalad to develop the prevesical space. A self-retaining Balfour retractor is placed in the incision to retract the rectus muscles laterally. The anterior bladder wall is identified, and two 2–0 Vicryl stitches are placed on each side of the midline below the peritoneal reflection. A vertical cystotomy is then made with an electrocautery and using a pair of Metzenbaum scissors, the cystotomy is extended cephalad and caudally to within 1 cm of the bladder neck. Several pairs of stay sutures are placed using 2–0 Vicryl on each side of the midline to facilitate exposure. A figure-of-eight suture using 0 Vicryl is placed and tied at the most caudal position of the cystotomy to prevent further extension of the cystotomy incision during blunt finger dissection of the adenoma. This suture is subsequently used to close the cystostomy as the first layer. After inspecting the bladder, a Millins retractor is placed in the bladder and used to further expose the trigone bringing the bladder neck and prostate into view. The ureteric orifices are identified and using electrocautery, a circular incision is made in the bladder mucosa distal to the trigone. Care is taken not to injure the ureteric orifices. With the use of a pair of Metzenbaum scissors, the plane between the prostatic adenoma and prostatic capsule is developed at the 6-o’clock position and once well-established plane is created posteriorly, the prostatic adenoma is enucleated using blunt dissection. At the apex, the prostatic urethra is transected using a pinch action of the two fingertips avoiding excessive traction so as to avoid avulsing the urethra and injuring the sphincteric mechanism. The prostatic adenoma is removed from the prostatic fossa. The prostatic fossa is then examined for discrete bleeding sites that are controlled with 3–0 vicryl suture ligatures. In addition, a 0-vicryl suture is used to place two figure-of-eight sutures to advance the bladder mucosa into the prostatic fossa at the 5-o’clock and 7-o’clock positions at the prostatovesical junction to ensure control of the main arterial blood supply to the prostate. These maneuvers lead to complete hemostasis.

A 22 or 24 -Fr 3-way Foley urethral catheter with a 30-mL balloon is passed through the urethra into the bladder and the cystotomy incision closed in two layers. The first layer of closure is performed using the figure-of-eight suture of 0 Vicryl that was placed and tied at the most caudal position of the cystotomy using it as a running suture. The previously placed 2–0 Vicryl stay-sutures are tied over the first layer of closure to complete the two-layer closure completing a watertight bladder closure. Thirty milliliters of saline is placed in the balloon to ensure that the catheter balloon remains in the bladder and does not retract into the prostatic fossa. The urethral catheter is irrigated to confirm a watertight closure and to verify that haemorrhage is minimal. A small wound drain is placed via a separate stab incision lateral to the bladder and exits the skin. The rectus fascia is closed with vicryl 1 suture in a running fashion. The skin is closed with nylon 3–0. The drain is then secured to the abdominal wall. Continuous bladder irrigation is initiated to prevent clot formation. The urethral catheter is removed on post operative day 7 and the patient discharged with the skin stitches removed on post operative day 10.

For the operative technique in the retro-pubic open prostatectomy, the patient is positioned supine with the table placed in a mild Trendelenburg position without extension. The suprapubic area is shaved, prepped, and draped maintaining sterility. A 22 or 24-Fr Foley urethral catheter with a 30-mL balloon is passed into the bladder and connected to a sterile closed drainage system, and the balloon is inflated with 30 mL of saline. For the patients with refractory retention of urine, the urethral catheter is maintained for the procedure. A lower midline incision from the umbilicus to the pubic symphysis is made and deepened through the subcutaneous tissue. The linea alba is incised and the rectus abdominis muscles separated in the midline. The transversalis fascia is incised sharply exposing the space of Retzius. The peritoneum is mobilized cephalad starting at the pubic symphysis. A self-retaining Balfour retractor is placed in the incision and widened and a well-padded, malleable blade is connected to the retractor and used to displace the bladder posteriorly and superiorly. The anterior surface of the bladder and prostate are exposed. The preprostatic adipose tissue is gently removed to expose the superficial branch of the dorsal vein complex and the puboprostatic ligaments with coagulation of the superficial branch of the dorsal vein. Next, we gain complete control of the dorsal vein complex as well as the lateral pedicles at the bladder neck, the main arterial blood supply to the prostate gland. This is achieved by firstly incising the endopelvic fascia laterally and partial transection of the puboprostatic ligaments. A 3–0 Monocryl suture on a 5/8-inch circle-tapered needle is passed in the avascular plane between the urethra and the dorsal vein complex at the apex of the prostate and tied. The lateral pedicles at the prostatovesical junction are ligated using figure-of-eight suture (vicryl 0) deep into the prostatovesical junction thus securing the main arterial blood supply to the prostate adenoma. With a sponge stick on the bladder neck to depress the bladder posteriorly, a No. 15 blade on a long handle is used to make a transverse capsulotomy in the prostate 2.0 cm distal to the bladder neck. The incision is deepened to thelevel of the adenoma and extended laterally in each direction to permit complete enucleation. A pair of Metzenbaum scissors is used to dissect the overlying prostatic capsule from the underlying prostatic adenoma. Once a well-defined plane is sufficiently developed, the index finger is inserted between the prostatic adenoma and the capsule to further develop the plane laterally and posteriorly allowing for enucleation and removal of the prostate adenoma with preservation of a strip of posterior prostatic urethra. The prostatic fossa is carefully inspected to ensure that all of the adenoma has been removed and that hemostasis is complete. A 22 or 24 -Fr, three-way Foley catheter with a 30-mL balloon is inserted through the anterior urethra and prostatic fossa into the bladder and the prostatic capsule closed using viryl 2–0 in two layers water tight with the sutures beginning laterally and meeting in the midline. Thirty milliliters of water is then placed in the balloon to ensure that the catheter balloon remains in the bladder and does not retract into the prostatic fossa. The bladder is then irrigated with saline to ensure continued hemostasis and to test the capsular closure for leakage. A suction drain is placed via a separate stab incision lateral to the prostate and bladder on one side to prevent hematoma and urinoma formation. The pelvis is irrigated with copious amounts of normal saline solution, and the rectus fascia is re-approximated with a size 1 vicryl suture. The skin is closed with interrupted nylon 3.0 sutures. The drain is secured to the abdominal wall, and traction applied to the catheter by placing gauze bandaged around the urethral catheter. Bladder irrigation is instituted until the urine become clear of blood. The urethral catheter is removed on post operative day 7 and the patient discharged with removal of the skin stitches on the 10^th^ post operative day.

The data collected and entered into a proforma included the pre-operative haemoglobin level(Hb), presence of co-morbidities and the case type (elective or emergency). Emergency prostatectomy was performed for patients who presented with severe haematuria due to BPH or sepsis from indwelling/stuck catheters as part of their resuscitation. The other parameters documented included the indication for the surgery, the anaesthetic method used, the systolic pressure at start of the operation, the status of the operating surgeon (i.e. consultant or resident), the duration of surgery, the operative prostate weight and the estimated blood loss. The requirement of blood transfusion including the units of blood transfused was documented.

The decision to transfuse blood intra-operatively was based on pallor of the mucous membranes, blood pressure instability and difficulty maintaining oxygen saturation intra-operative and/or haemoglobin levels determined by using the HemoCue. For the post-operative assessment of the haemoglobin level, Standard laboratory determined haemoglobin levels using a BC-6800 hematology Analyzer by Mindray was used with haemoglobin level less than 8.0 g/dl serving as a trigger for blood transfusion post operatively. The enucleated prostate specimens were submitted for histopathological examination.

The data was analyzed using the statistical package for the social sciences (SPSS) version 21 with the results presented as percentages and mean with standard deviation. Categorical variables were analyzed by chi-square. A significant statistical difference was accepted whenever *p* < .05. Blood transfusion and pre-operative haemoglobin, were evaluated as categorical data.

A multivariable logistic regression analysis was performed using the variables pre-operative haemoglobin level, duration of surgery, age, ASA score, systolic blood pressure, type of anaesthesia, status of the operating surgeon and operative prostate weight.

## Results

### Patient characteristics

Two hundred patients who had open prostatectomy for BPH were studied.

The majority of the patients 136 (68.0 %) had refractory retention of urine as the indication for surgery (Table 1).

**Table 1:** Indications for open prostatectomy

Indication for open prostatectomy	Number of patients (%)
Refractory retention of urine	136 (68.0)
Haematuria due to BPH	45 (22.5)
Lower urinary tract symptoms	10 (5.0)
BPH with associated bladder calculi	4 (2.0)
BPH with associated mild renal dysfunction	2 (1.0)
Refractory retention of urine with stuck urethral catheter	3 (1.5)
Total	200 (100)

The mean age of the patients was 69.1 ± 9.1 year (range 48–92 years) with mean body weight of 70.8 ± 12.6 kg. The mean pre-operative haemoglobin level was 12.0 ± 2.4 g/dl and that of the systolic blood pressure at the start of surgery was 152.2 ± 21.8 mmHg (range 102–219 mmHg). The mean surgery time (duration of Surgery) was 101.3 ± 32.5mins with that of the estimated blood loss being 365.8 ± 226.1 mls. The mean operative prostate weight was 110.5 ± 90.5 g (Table 2).

**Table 2:** Patients characteristics

	Age (yrs)	Pre-operative Hb (g/dl)	Systolic Blood pressure (mmHg)	Surgery Time (duration of surgery) [mins]	Estimated blood loss (ml)	Operative prostate weight (g)
Mean	69.0859	11.9943	152.1632	101.3100	365.7895	110.5094
Median	69.0000	12.6000	150.0000	95.0000	300.0000	80.0000
Mode	75.00	13.30	140.00(a)	90.00	300.00	60.00
Std. Deviation	9.14562	2.36919	21.75062	32.49003	226.11000	90.45363
Range	44.00	13.30	117.00	205.00	900.00	535.00
Minimum	48.00	3.10	102.00	35.00	100.00	15.00
Maximum	92.00	16.40	219.00	240.00	1000.00	550.00

Ninety (45 %) of the patients had associated co-morbidities comprising hypertension 75 (37.5 %) [Including one person with hypertension and asthma], diabetes mellitus alone 4 (2.0 %), hypertension with diabetes mellitus 10 (5.0 %) and bronchial asthma 1 (0.5 %).

One hundred and sixty-seven (83.5 %) of the cases were elective cases while 33 (16.5 %) were emergencies.

Eighty-eight of the surgeries (44.0 %) were performed by senior residents in urology while 112 (56.0 %) were carried out by consultant urologists. Open transvesical prostatectomy was the operative method used in 180 (90.0 %) of the cases whilst retropubic prostatectomy was in 20 (10.0 %). Spinal, general and epidural anaesthesia were used in 164 (82.0 %), 28 (14.0 %) and 8 (4.0 %) of the operations respectively. Forty-seven (23.5 %) 0f the cases were transfused while 153 (76.5 %) were not (Table 3).

**Table 3:** Patients characteristics (categories)

VARIABLE	Number of patients	%
Blood Transfusion		
Transfused	47	23.5
Not-transfused	153	76.5
Age (*n* = 198)		
≤70 yrs	109	55.1
>70 yrs	89	44.9
Co-Morbidities		
No comorbidities	110	55.0
Presence of comorbidities	90	45.0
Case Type		
Elective	167	83.5
Emergency	33	16.5
Status of Surgeon		
Consultant	112	56.0
Resident	88	44.0
Anaestheisa used		
Spinal anesthesia	164	82.0
General anaesthesia	28	14.0
Epidural	8	4.0
Operative Method used		
Transvesical prostatectomy	180	90.0
Millins retropubic prostatectomy	20	10.0
Operative Prostate weight		
Prostate weight ≤ 100 g	130	65.0
Prostate weight >100 g	70	35.0
Duration of surgery		
Surgery time ≤ 90mins	95	47.5
Surgery time > 90mins	105	52.5
Total	200	

A total of 95 units of blood were transfused with an overall transfusion rate of 23.5 % (47/200) (Table 4).

**Table 4:** Number of units of blood transfused

Units of blood transfused	Number of patients (%)
0	153(76.5)
1	13 (6.5)
2	25 (12.5)
3	5 (2.5)
≥4	4 (2.0)
Total	200 (100)

After histological review of the enucleated prostate specimen, two cases (1 %) had foci of adenocarcinoma but the rest were confirmed as benign prostate hyperplasia. None of the patients with foci of adenocarcinoma was transfused.

### Relationship between the blood transfusion rate and various patient characteristics

Certain individual parameters had varied influence on the transfusion rate. The transfusion rate was significantly higher in patients with anaemia (*p* = .000), emergency cases (*p* = .000), the use of general anaesthesia (*p* = .002), a resident as the operating surgeons (*p* = .034), prostate weight > 100 g (*p* = .000) and duration of surgery (*p* = .011) (Tables 5, 6 and 7).

**Table 5:** Blood transfusion against age, systolic blood pressure and enucleated prostate weight

			Blood Transfusion		Total	*p*-value
			Not-transfused	Transfused		
Patient age	Age ≤ 70 yrs	Count	83	26	109	
		% within transfusion	55.0 %	55.3 %	55.1 %	
	Age >70 yrs	Count	68	21	89	
		% within transfusion	45.0 %	44.7 %	44.9 %	
Total		Count	151	47	198	0.551
Systolic blood pressure	Systolic blood pressure ≤ 140 mmHg	Count	56	17	73	
		% within transfusion	36.6 %	36.2 %	36.5 %	
	Systolic blood pressure > 140 mmHg	Count	97	30	127	
		% within transfusion	63.4 %	63.8 %	63.5 %	
Total		Count	153	47	200	0.551
Enucleated Prostate weight	Prostate weight ≤ 100 g	Count	111	19	130	
		% within transfusion	72.5 %	40.4 %	65.0 %	
	Prostate weight > 100 g	Count	42	28	70	
		% within transfusion	27.5 %	59.6 %	35.0 %	
Total		Count	153	47	200	
		% within transfusion	100.0 %	100.0 %	100.0 %	0.000

**Table 6:** Blood transfusion against Hb, case type and co-morbidities

			Blood Transfusion		Total	*p*-value
			Not-transfused	Transfused		
Hb Level	Severe Anaemia (Hb < 8.0 g/dl)	Count	1	12	13	
		% within transfusion	.7 %	25.5 %	6.5 %	
	Moderate Anaemia (Hb 8.0–10.9 g/dl)	Count	23	21	44	
		% within transfusion	15.0 %	44.7 %	22.0 %	
	Mild Anaemia (11.0–12.9 g/dl)	Count	43	9	52	
		% within transfusion	28.1 %	19.1 %	26.0 %	
	Normal (≥13.0 g/dl)	Count	86	5	91	
		% within transfusion	56.2 %	10.6 %	45.5 %	
Total		Count	153	47	200	0.000
Case type	Elective	Count	143	24	167	
		% within transfusion	93.5 %	51.1 %	83.5 %	
	Emergency	Count	10	23	33	
		% within transfusion	6.5 %	48.9 %	16.5 %	
Total		Count	153	47	200	0.000
Co-morbidities	No co-morbidity	Count	76	34	110	
		% within transfusion	49.7 %	72.3 %	55.0 %	
	Hypertension	Count	66	9	75	
		% within transfusion	43.1 %	19.1 %	37.5 %	
	Diabetes Mellitus	Count	3	1	4	
		% within transfusion	2.0 %	2.1 %	2.0 %	
	Hypertension with diabetes Mellitus	Count	7	3	10	
		% within transfusion	4.6 %	6.4 %	5.0 %	
	Asthma	Count	1	0	1	
		% within transfusion	.7 %	0.0 %	.5 %	
Total		Count	153	47	200	
		% within transfusion	100.0 %	100.0 %	100.0 %	0.051

**Table 7:** Blood transfusion against type of anaesthesia, status of surgeon, operative method and duration of surgery

			Blood Transfusion		Total	*p*-value
			Not-transfused	Transfused		
Anesthesia	Spinal	Count	132	32	164	
		% within transfusion	86.3 %	68.1 %	82.0 %	
	Ga	Count	14	14	28	
		% within transfusion	9.2 %	29.8 %	14.0 %	
	Epidural	Count	7	1	8	
		% within transfusion	4.6 %	2.1 %	4.0 %	
Total		Count	153	47	200	0.002
Status of operating surgeon	Consultant	Count	92	20	112	
		% within transfusion	60.1 %	42.6 %	56.0 %	
	Resident	Count	61	27	88	
		% within transfusion	39.9 %	57.4 %	44.0 %	
Total		Count	153	47	200	0.034
Operative method	Transvesical	Count	136	44	180	
		% within transfusion	88.9 %	93.6 %	90.0 %	
	Retropubic	Count	17	3	20	
		% within transfusion	11.1 %	6.4 %	10.0 %	
Total		Count	153	47	200	
		% within transfusion	100.0 %	100.0 %	100.0 %	0.345
Duration of Surgery	Surgery time ≤ 90mins	Count	80	15	95	
		% within transfusion	52.3 %	31.9 %	47.5 %	
	Surgery time > 90mins	Count	73	32	105	
		% within transfusion	47.7 %	68.1 %	52.5 %	
Total		Count	153	47	200	
		% within transfusion	100.0 %	100.0 %	100.0 %	0.011

For patients with severe anaemia (pre-operative blood haemoglobin level <8.0 g/dl), the transfusion rate was 92.3 % while the transfusion rate for those with normal pre-operative blood haemoglobin level ≥ 13.0 g/dl was 5.5 % (Table 6). The patients with Hb < 8 that were not transfused were emergency cases who underwent the procedure without blood being available in the blood bank. They however kept their haemodynamic stability after the procedure and hence no further transfusions were offered them.

### Multivariable logistic regression

In a multivariable logistic regression analysis using the variables pre-operative haemoglobin level (evaluated as categorical), duration of surgery, age, ASA score, systolic blood pressure, type of anaesthesia, status of the operating surgeon and operative prostate weight, only the pre-operative haemoglobin level(as categorical data) (*p* = .000, Odds Ratio (OR) = 0.95, 95 % Confidence Interval (CI) [0.035–0.257]) and duration of surgery (as a continuous data) (*p* = .025, Odds Ratio (OR) = 1.021, 95 % Confidence Interval (CI) [1.003–1.039]) could predict the likelihood of blood transfusion in open prostatectomy for BPH (Table 8).

**Table 8:** Multivariable logistic regression analysis

Variables in the Equation								
Predictor Variables	B	S.E.	Wald (chi-square)	df	*p*-value	odd ratio	95 % C.I.for odd ratio	
							Lower	Upper
Pre-operative Hb (Anaemia)	−2.352	.507	21.517	1	.000	.095	.035	.257
Surgery time (Duration of surgery)	.020	.009	5.021	1	.025	1.021	1.003	1.039
Age category	−.212	.616	.119	1	.730	.809	.242	2.705
ASA score	−.369	.475	.605	1	.437	.691	.273	1.753
Systolic blood pressure	−.120	.334	.128	1	.720	.887	.461	1.708
Anesthesia	.585	.648	.814	1	.367	1.794	.504	6.390
Status of operating surgeon	1.039	.618	2.828	1	.093	2.827	.842	9.489
Operative prostate weight	.003	.004	.524	1	.469	1.003	.996	1.010
Constant	1.651	2.384	.479	1	.489	5.211		

Therefore, the multivariable logistic regression is given by

Log (transfused) = 1.651 – 2.352X1 + 0.020X2 – 0.212X3 – 0.369X4 – 0.120X5 + 0.585X6 + 1.039X7 + 0.003X8

Variable(s) entered on step 1: hb (anaemia), surgery time, age category, ASA score, systolic, anaesthesia, status of surgeon, operative prostate weight

## Discussion

Peri-operative blood transfusion is common in open prostatectomy for BPH and that remains a significant disadvantage. The reported peri-operative blood transfusion rate has ranged widely from 3.3 % to 36.8 % being 3.3 % in a report by Zargooshi J (Iran) [6], 24.5 % by Elshai et al (Egypt) [7] and 36.8 % by Ngugi et al (Kenya) [8]. The blood transfusion rate of 23.5 % in the present study compares with some of these reports.

Different factors have been reported to play a role in determining the need for blood transfusion in open prostatectomy for BPH. Previous reports have indicated that patient factors such as age above 70 years, increasing ASA scores and a systolic blood pressure above 140 mmHg might contribute to the need for blood transfusion in open prostatectomy [8]. These factors were not observed to be significant in this study. Of interest though is the finding of an increased transfusion rate in ASA3 by Torres-Claramunt R et al in a study on the predictors of blood transfusion in patients undergoing elective surgery for degenerative diseases of the spine [13].

This study found the presence of anaemia to be associated with a significantly higher blood transfusion rate. Using the WHO definition for anaemia, (http://www.who.int/vmnis/indicators/haemoglobin.pdf) patients who had severe anaemia pre-operatively were more likely to be transfused compared with those with normal blood haemoglobin levels. (*p* = .000). Hence the pre-operative blood haemoglobin level should serve as a guide to possible need for blood transfusion in open prostatectomy for BPH. Improving the blood haemoglobin level to normal levels before surgery could probably reduce the blood transfusion rate for this operation. The significantly higher blood transfusion rate in emergency cases may be partly due to inadequate pre-operative preparation before the procedure is undertaken in these rather ill patients. Transfusion rate of 14.4 % in the elective cases is comparable to that of 8.2 % reported by Serrata et al. [14].

There was an observed lower blood transfusion rate in patients with co-morbidities as compared to patients without any co-morbidities (*p* = .051). The reason for this was not obvious. A possible explanation could be that those with co-morbidities had a more rigorous pre-operative preparation before surgery.

A significantly higher blood transfusion rate was observed in the operations performed under general anaesthesia. (*p* = .002) It is noteworthy that an increased use of blood transfusion associated with general anaesthesia has been observed in relation to total hip arthroplasty by Maurer et al. [15].

Although no significant difference was found between the two standard operative techniques for open prostatectomy in this study (*p* = .345), Dall’Oglio et al reported a reduced transfusion rate in an improved technique of Millin’s retropubic prostatectomy compared to a classical transvesical prostatectomy [16].

The availability of surgeons specially trained in the procedure of open prostatectomy for BPH is not uniform across countries and even within a particular country. In some developing countries open prostatectomy is carried out by general surgeons [5] and in others, a significant proportion of the procedures are done by residents in training [7]. The attendant differences in experience and expertise of these categories of surgeons in performing open prostatectomy may partly be responsible for the wide variation in the reported transfusion rates for the procedure from different parts of the world. The present study showed that in our centre, the blood transfusion rate was significantly higher in the residents with less experience compared to that of the consultants with more experience in performing this surgery (30.7 % vrs 17.9 % [*p* = .034].

The finding of a significant association between the blood transfusion rate and the duration of surgery is indicative of an increased likelihood of transfusion with a longer duration of surgery. Comparing the blood transfusion rate and the mean prostate weight in various series showed no pattern of an increased blood transfusion with increasing mean prostate weights. Blood transfusion rates of 12.7 %, 6.8 %, 36.8 % and 8.2 % have been reported in procedures with mean prostate weights of 88.7 g, 104.5 g, 66.9 g and 75 g respectively [1, 4, 8, 14]. In this study a blood transfusion rate of 23.5 % was observed corresponding to a mean prostate weight of 110.5 g. Suer et al reported an increased blood transfusion rate of 19.2 % for prostates greater than 100 g compared to those with prostate weight less than 100 g (9.4 %) [4]. Elshai et al upon stratification found no increase in blood transfusion rates between prostate weights greater than or less than 120 g [7]. This study confirmed the findings of Suer et al as the blood transfusion rate was significantly higher in prostates > 100 g (40.0 %) compared with prostate weights ≤ 100.0 g (14.6 %) [*p* = .000]. Even though there are currently methods for surgical resection of large prostates such as HoLEP, ThuLRP and PVP which have been found safe and effective and probable requiring less blood transfusion, [3] open prostatectomy is still used for large prostates in low resource countries like Ghana due to unavailability of these other treatment modalities.

Of the various factors which were found to be associated significantly with increased blood transfusion rate, only the pre-operative blood haemoglobin level (as categorical data) (*p* = .000, OR 0.95, 95 % CI [0.035–0.257]) and the duration of surgery (as continuous data) (*p* = .025, OR 1.02, 95 % CI[1.003–1.039]) could predict the likelihood of blood transfusion in open prostatectomy for BPH following a multivariable logistic regression analysis.

## Conclusions

Various factors were found to be associated significantly with increased blood transfusion rate in open prostatectomy for BPH. These included the presence of anaemia, emergency cases, use of general anaesthesia, prostate weight > 100 g, duration of surgery and a resident as the operating surgeon. However, only the pre-operative blood haemoglobin level and the duration of surgery could predict the likelihood of blood transfusion in open prostatectomy for BPH.

For patients with normal blood haemoglobin level (Hb ≥ 13.0 g/dl), a ‘group and save’ policy should be the preferred blood ordering procedure if undergoing an elective open prostatectomy for BPH, which preferably should be performed under spinal anaesthesia.
